# Surgeons’ perspectives on artificial intelligence to support clinical decision-making in trauma and emergency contexts: results from an international survey

**DOI:** 10.1186/s13017-022-00467-3

**Published:** 2023-01-03

**Authors:** Lorenzo Cobianchi, Daniele Piccolo, Francesca Dal Mas, Vanni Agnoletti, Luca Ansaloni, Jeremy Balch, Walter Biffl, Giovanni Butturini, Fausto Catena, Federico Coccolini, Stefano Denicolai, Belinda De Simone, Isabella Frigerio, Paola Fugazzola, Gianluigi Marseglia, Giuseppe Roberto Marseglia, Jacopo Martellucci, Mirko Modenese, Pietro Previtali, Federico Ruta, Alessandro Venturi, Haytham M. Kaafarani, Tyler J. Loftus, Team Dynamics Study Group, Kenneth Lyle Abbott, Abubaker Abdelmalik, Nebyou Seyoum Abebe, Fikri Abu-Zidan, Yousif Abdallah Yousif Adam, Harissou Adamou, Dmitry Mikhailovich Adamovich, Ferdinando Agresta, antonino Agrusa, Emrah Akin, Mario Alessiani, Henrique Alexandrino, Syed Muhammad Ali, Vasilescu Alin Mihai, Pedro Miguel Almeida, Mohammed Mohammed Al-Shehari, Michele Altomare, Francesco Amico, Michele Ammendola, Jacopo Andreuccetti, Elissavet Anestiadou, Peter Angelos, Alfredo Annicchiarico, Amedeo Antonelli, Daniel Aparicio-Sanchez, antonella Ardito, Giulio Argenio, Catherine Claude Arvieux, Ingolf Harald Askevold, Boyko Tchavdarov Atanasov, Goran Augustin, Selmy Sabry Awad, Giulia Bacchiocchi, Carlo Bagnoli, Hany Bahouth, Efstratia Baili, Lovenish Bains, Gian Luca Baiocchi, Miklosh Bala, Carmen Balagué, Dimitrios Balalis, Edoardo Baldini, oussama Baraket, Suman Baral, Mirko Barone, Alberto Gonzãlez Barranquero, Jorge Arturo Barreras, Gary Alan Bass, Zulfu Bayhan, Giovanni Bellanova, Offir Ben-Ishay, Fabrizio Bert, Valentina Bianchi, Helena Biancuzzi, Chiara Bidoli, Raluca Bievel Radulescu, Mark Brian Bignell, Alan Biloslavo, Roberto Bini, Paolo Boati, Guillaume Boddaert, Branko Bogdanic, Cristina Bombardini, Luigi Bonavina, Luca Bonomo, Andrea Bottari, Konstantinos Bouliaris, Gioia Brachini, Antonio Brillantino, Giuseppe Brisinda, Maloni Mamada Bulanauca, Luis Antonio Buonomo, Jakob Burcharth, Salvatore Buscemi, Francesca Calabretto, Giacomo Calini, Valentin Calu, Fabio Cesare Campanile, Riccardo Campo Dall′Orto, Andrea Campos-Serra, Stefano Campostrini, Recayi Capoglu, Joao Miguel Carvas, Marco Cascella, Gianmaria Casoni Pattacini, Valerio Celentano, Danilo Corrado Centonze, Marco Ceresoli, Dimitrios Chatzipetris, Antonella Chessa, Maria Michela Chiarello, Mircea Chirica, Serge Chooklin, Christos Chouliaras, Sharfuddin Chowdhury, Pasquale Cianci, Nicola Cillara, Stefania Cimbanassi, Stefano Piero Bernardo Cioffi, Elif Colak, Enrique Colás Ruiz, Luigi Conti, Alessandro Coppola, Tiago Correia De Sa, Silvia Dantas Costa, Valerio Cozza, Giuseppe Curro’, Kirsten Felicia Ann-Sophie Aimee Dabekaussen, Fabrizio D’Acapito, Dimitrios Damaskos, Giancarlo D’Ambrosio, Koray Das, Richard Justin Davies, Andrew Charles De Beaux, Sara Patricia De Lebrusant Fernandez, Alessandro De Luca, Francesca De Stefano, Luca Degrate, Zaza Demetrashvili, Andreas Kyriacou Demetriades, Dzemail Smail Detanac, Agnese Dezi, Giuseppe Di Buono, Isidoro Di Carlo, Pierpaolo Di Lascio, Marcello Di Martino, Salomone Di Saverio, Bogdan Diaconescu, Jose J. Diaz, Rigers Dibra, Evgeni Nikolaev Dimitrov, Vincenza Paola Dinuzzi, Sandra Dios-Barbeito, Jehangir Farman Ali Diyani, Agron Dogjani, Maurizio Domanin, Mario D’Oria, Virginia Duran Munoz-Cruzado, Barbora East, Mikael Ekelund, Gerald Takem Ekwen, Adel Hamed Elbaih, Muhammed Elhadi, Natalie Enninghorst, Mairam Ernisova, Juan Pablo Escalera-Antezana, Sofia Esposito, Giuseppe Esposito, Mercedes Estaire, Camilla Nikita Farè, Roser Farre, Francesco Favi, Luca Ferrario, Antonjacopo Ferrario di Tor Vajana, Claudia Filisetti, Francesco Fleres, Vinicius Cordeiro Fonseca, Alexander Forero-Torres, Francesco Forfori, Laura Fortuna, Evangelos Fradelos, Gustavo P. Fraga, Pietro Fransvea, Simone Frassini, Giuseppe Frazzetta, Erica Pizzocaro, Maximos Frountzas, Mahir Gachabayov, Rita Galeiras, Alain A. Garcia Vazquez, Simone Gargarella, Ibrahim Umar Garzali, Wagih Mommtaz Ghannam, Faiz Najmuddin Ghazi, Lawrence Marshall Gillman, Rossella Gioco, Alessio Giordano, Luca Giordano, Carlo Giove, Giorgio Giraudo, Mario Giuffrida, Michela Giulii Capponi, Emanuel Gois, Carlos Augusto Gomes, Felipe Couto Gomes, Ricardo Alessandro Teixeira Gonsaga, Emre Gonullu, Jacques Goosen, Tatjana Goranovic, Raquel Gracia-Roman, Giorgio Maria Paolo Graziano, Ewen Alexander Griffiths, Tommaso Guagni, Dimitar Bozhidarov Hadzhiev, Muad Gamil Haidar, Hytham K. S. Hamid, Timothy Craig Hardcastle, Firdaus Hayati, Andrew James Healey, Andreas Hecker, Matthias Hecker, Edgar Fernando Hernandez Garcia, Adrien Montcho Hodonou, Eduardo Cancio Huaman, Martin Huerta, Aini Fahriza Ibrahim, Basil Mohamed Salabeldin Ibrahim, Giuseppe Ietto, Marco Inama, Orestis Ioannidis, Arda Isik, Nizar Ismail, Azzain Mahadi Hamid Ismail, Ruhi Fadzlyana Jailani, Ji Young Jang, Christos Kalfountzos, Sujala Niatarika Rajsain Kalipershad, Emmanouil Kaouras, Lewis Jay Kaplan, Yasin Kara, Evika Karamagioli, Aleksandar Karamarkovia, Ioannis Katsaros, Alfie J. Kavalakat, Aristotelis Kechagias, Jakub Kenig, Boris Juli Kessel, Jim S. Khan, Vladimir Khokha, Jae Il Kim, Andrew Wallace Kirkpatrick, Roberto Klappenbach, Yoram Kluger, Yoshiro Kobe, Efstratios Kofopoulos Lymperis, Kenneth Yuh Yen Kok, Victor Kong, Dimitris P. Korkolis, Georgios Koukoulis, Bojan Kovacevic, Vitor Favali Kruger, Igor A. Kryvoruchko, Hayato Kurihara, Akira Kuriyama, Aitor Landaluce-Olavarria, Pierfrancesco Lapolla, Ari Leppäniemi, Leo Licari, Giorgio Lisi, Andrey Litvin, Aintzane Lizarazu, Heura Llaquet Bayo, Varut Lohsiriwat, Claudia Cristina Lopes Moreira, Eftychios Lostoridis, Agustãn. Tovar Luna, Davide Luppi, Gustavo Miguel Machain V., Marc Maegele, Daniele Maggiore, Stefano Magnone, Ronald V. Maier, Piotr Major, Mallikarjuna Manangi, andrea manetti, Baris Mantoglu, Chiara Marafante, Federico Mariani, Athanasios Marinis, Evandro Antonio Sbalcheiro Mariot, Gennaro Martines, Aleix Martinez Perez, Costanza Martino, Pietro Mascagni, Damien Massalou, Maurizio Massaro, Belen Matías-García, Gennaro Mazzarella, Giorgio Mazzarolo, Renato Bessa Melo, Fernando Mendoza-Moreno, Serhat Meric, Jeremy Meyer, Luca Miceli, Nikolaos V. Michalopoulos, Flavio Milana, Andrea Mingoli, Tushar S. Mishra, Muyed Mohamed, Musab Isam Eldin Abbas Mohamed, Ali Yasen Mohamedahmed, Mohammed Jibreel Suliman Mohammed, Rajashekar Mohan, Ernest E. Moore, Dieter Morales-Garcia, MÃ¥ns Muhrbeck, Francesk Mulita, Sami Mohamed Siddig Mustafa, Edoardo Maria Muttillo, Mukhammad David Naimzada, Pradeep H. Navsaria, Ionut Negoi, Luca Nespoli, Christine Nguyen, Melkamu Kibret Nidaw, Giuseppe Nigri, Ioannis Nikolopoulos, Donal Brendan O’Connor, Habeeb Damilola Ogundipe, Cristina Oliveri, Stefano Olmi, Ernest Cun Wang Ong, Luca Orecchia, Aleksei V. Osipov, Muhammad Faeid Othman, Marco Pace, Mario Pacilli, Leonardo Pagani, Giuseppe Palomba, Desire’ Pantalone, Arpad Panyko, Ciro Paolillo, Mario Virgilio Papa, Dimitrios Papaconstantinou, Maria Papadoliopoulou, Aristeidis Papadopoulos, Davide Papis, Nikolaos Pararas, Jose Gustavo Parreira, Neil Geordie Parry, Francesco Pata, Tapan Patel, Simon Paterson-Brown, Giovanna Pavone, Francesca Pecchini, Veronica Pegoraro, Gianluca Pellino, Maria Pelloni, Andrea Peloso, Eduardo Perea Del Pozo, Rita Goncalves Pereira, Bruno Monteiro Pereira, Aintzane Lizarazu Perez, Silvia Pérez, Teresa Perra, Gennaro Perrone, Antonio Pesce, Lorenzo Petagna, Giovanni Petracca, Vorapong Phupong, Biagio Picardi, Arcangelo Picciariello, Micaela Piccoli, Edoardo Picetti, Emmanouil Pikoulis Pikoulis, Tadeja Pintar, Giovanni Pirozzolo, Francesco Piscioneri, Mauro Podda, Alberto Porcu, Francesca Privitera, Clelia Punzo, Silvia Quaresima, Martha Alexa Quiodettis, Niels Qvist, Razrim Rahim, Filipe Ramalho de Almeida, Rosnelifaizur Bin Ramely, Huseyin Kemal Rasa, Martin Reichert, Alexander Reinisch-Liese, Angela Renne, Camilla Riccetti, Maria Rita Rodriguez-Luna, Daniel Roizblatt, Andrea Romanzi, Luigi Romeo, Francesco Pietro Maria Roscio, Ramely Bin Rosnelifaizur, Stefano Rossi, Andres M. Rubiano, Elena Ruiz-Ucar, Boris Evgeniev Sakakushev, Juan Carlos Salamea, Ibrahima Sall, Lasitha Bhagya Samarakoon, Fabrizio Sammartano, Alejandro Sanchez Arteaga, Sergi Sanchez-Cordero, Domenico Pietro Maria Santoanastaso, Massimo Sartelli, Diego Sasia, NORIO SATO, Artem Savchuk, Robert Grant Sawyer, Giacomo Scaioli, DIMITRIOS SCHIZAS, Simone Sebastiani, Barbara Seeliger, Helmut Alfredo Segovia Lohse, Charalampos Seretis, Giacomo Sermonesi, Mario Serradilla-Martin, Vishal G. Shelat, Sergei Shlyapnikov, Theodoros Sidiropoulos, Romeo Lages Simoes, Leandro Siragusa, Boonying Siribumrungwong, Mihail Slavchev, Leonardo Solaini, gabriele soldini, Andrey Sopuev, Kjetil Soreide, APOSTOLOS SOVATZIDIS, Philip Frank Stahel, Matt Strickland, Mohamed Arif Hameed Sultan, Ruslan Sydorchuk, Larysa Sydorchuk, Syed Muhammad Ali Muhammad Syed, Luis Tallon-Aguilar, Andrea Marco Tamburini, Nicolò Tamini, Edward C. T. H. Tan, Jih Huei Tan, Antonio Tarasconi, Nicola Tartaglia, Giuseppe Tartaglia, Dario Tartaglia, John Vincent Taylor, Giovanni Domenico Tebala, Ricardo Alessandro Teixeira Gonsaga, Michel Teuben, Alexis Theodorou, Matti Tolonen, Giovanni Tomasicchio, Adriana Toro, Beatrice Torre, Tania Triantafyllou, Giuseppe Trigiante Trigiante, Marzia Tripepi, Julio Trostchansky, Konstantinos Tsekouras, Victor Turrado-Rodriguez, Roberta Tutino, Matteo Uccelli, Petar Angelov Uchikov, Bakarne Ugarte-Sierra, Mika Tapani Ukkonen, Michail Vailas, Panteleimon G. Vassiliu, Alain Garcia Vazquez, Rita Galeiras Vazquez, George Velmahos, Juan Ezequiel Verde, Juan Manuel Verde, Massimiliano Veroux, Jacopo Viganò, Ramon Vilallonga, Diego Visconti, Alessandro Vittori, Maciej Waledziak, Tongporn Wannatoop, Lukas Werner Widmer, Michael Samuel James Wilson, Sarah Woltz, Ting Hway Wong, Sofia Xenaki, Byungchul Yu, Steven Yule, Sanoop Koshy Zachariah, Georgios Zacharis, Claudia Zaghi, Andee Dzulkarnaen Zakaria, Diego A. Zambrano, Nikolaos Zampitis, Biagio Zampogna, Simone Zanghì, Maristella Zantedeschi, Konstantinos Zapsalis, Fabio Zattoni, Monica Zese

**Affiliations:** 1 Department of Clinical, Diagnostic and Pediatric Sciences, University of Pavia, Via Alessandro Brambilla, 74, 27100 Pavia, PV Italy; 2 General Surgery, IRCCS Policlinico San Matteo Foundation, Pavia, Italy; 3 ITIR – Institute for Transformative Innovation Research, University of Pavia, Pavia, Italy; 4 Department of Neurosurgery, ASUFC Santa Maria Della Misericordia, Udine, Italy; 5 Department of Management, Ca’ Foscari University of Venice, Venice, Italy; 6 Bufalini Hospital, AUSL Romagna, Cesena, Italy; 7 Department of Surgery, University of Florida Health, Gainesville, FL USA; 8 Division of Trauma and Acute Care Surgery, Scripps Memorial Hospital La Jolla, La Jolla, CA USA; 9 Department of HPB Surgery, Pederzoli Hospital, Peschiera del Garda, Italy; 10 General, Emergency and Trauma Surgery Department, Pisa University Hospital Pisa, Pisa, Italy; 11 Department of Economics and Management, University of Pavia, Pavia, Italy; 12 Department of Emergency, Digestive and Metabolic Minimally Invasive Surgery, Poissy and Saint Germain en Laye Hospitals, Poissy, France; 13 IRCCS Policlinico San Matteo Foundation, Pediatric Clinic., Pavia, Italy; 14 Department of Civil Engineering and Architecture, University of Pavia, Pavia, Italy; 15 Department of Surgery, Careggi University Hospital, Florence, Italy; 16 Humco Srl, Venice, Italy; 17 General Direction, ASL BAT (Health Agency), Andria, Italy; 18 Department of Political and Social Sciences, University of Pavia, Pavia, Italy; 19 Bureau of the Presidency, IRCCS Policlinico San Matteo Foundation, Pavia, Italy; 20 Harvard Medical School, Boston, MA USA; 21 Division of Trauma, Emergency Surgery, and Surgical Critical Care, Massachusetts General Hospital, Boston, MA USA

**Keywords:** Artificial intelligence, Clinical decision-making, Decision aids, Trauma and emergency surgery, Survey

## Abstract

**Background:**

Artificial intelligence (AI) is gaining traction in medicine and surgery. AI-based applications can offer tools to examine high-volume data to inform predictive analytics that supports complex decision-making processes. Time-sensitive trauma and emergency contexts are often challenging. The study aims to investigate trauma and emergency surgeons’ knowledge and perception of using AI-based tools in clinical decision-making processes.

**Methods:**

An online survey grounded on literature regarding AI-enabled surgical decision-making aids was created by a multidisciplinary committee and endorsed by the World Society of Emergency Surgery (WSES). The survey was advertised to 917 WSES members through the society’s website and Twitter profile.

**Results:**

650 surgeons from 71 countries in five continents participated in the survey. Results depict the presence of technology enthusiasts and skeptics and surgeons' preference toward more classical decision-making aids like clinical guidelines, traditional training, and the support of their multidisciplinary colleagues. A lack of knowledge about several AI-related aspects emerges and is associated with mistrust.

**Discussion:**

The trauma and emergency surgical community is divided into those who firmly believe in the potential of AI and those who do not understand or trust AI-enabled surgical decision-making aids. Academic societies and surgical training programs should promote a foundational, working knowledge of clinical AI.

## Background

Artificial intelligence (AI) is defined as the development of algorithms that give machines the ability to act with human-like rationality in complex tasks, such as problem-solving and decision-making, and is poised to reshape medicine and surgery broadly [1].

Diagnostic and judgment errors are the second leading cause of preventable harm [2]. Decision-making is one of the most challenging and crucial tasks performed by trauma and emergency surgeons, where time constraints and high cognitive loads from high volumes of information demand reliance on cognitive shortcuts, leading to mistakes and preventable patient harm [3].

In recent years, decision support systems based on AI algorithms are receiving considerable attention in the literature [4–6]. In contrast to human cognitive capacities, AI can be used to examine high-complexity and high-volume data for predictive analytics to augment the precision of complex decision-making processes. Nonetheless, deploying medical AI systems in routine trauma and emergency surgery care presents a critical yet largely unfulfilled opportunity as the medical AI community steers the complex ethical, technical, and human-centered challenges required for safe and adequate translation [7–10]. Experienced surgeons are appropriately cautious of enthusiastic, novel solutions that are unsubstantiated by academic rigor and evidence of performance advantages in clinical settings [8]. Even when AI accurately predicts clinical outcomes hours or days in advance, its application in trauma and emergency surgery will remain marginal until the deployment proves trust in the accuracy and the lack of bias of the model, addresses target risk-sensitive decisions, and integrates with clinical workflows [11, 12].

The recently published artificial intelligence in Emergency and Trauma Surgery (ARIES) survey, endorsed by the World Society of Emergency Surgery (WSES) evaluated the knowledge, attitude, and practices in the application of AI in the emergency setting among international acute care and emergency surgeons. The investigation revealed how the implementation of AI in the emergency and trauma setting is highly anticipated but still in an early phase, with trauma and emergency surgeons enthusiastic about being involved [13].

Starting from these premises and research gaps, this article aims to assess surgeons’ understanding and knowledge about the role of new technologies like AI in clinical decision-making by employing an international survey endorsed by the WSES.

## Methods

### Design and setting

The exploratory study of the international trauma and emergency surgeons’ community used a population-based online questionnaire to gather demographic, knowledge, and practice-based information regarding the adoption of new technological tools to support clinical decision-making. The online questionnaire was generated in English through Google Forms [14, 15], and followed the Checklist for Reporting Results of Internet E-Surveys (CHERRIES) [16].

A steering committee within the WSES was appointed, involving a multidisciplinary panel of academics and practitioners in the fields of trauma and emergency surgery, healthcare management and policies, innovation, business and medical ethics, information technology, law, and organization science. No Institutional Review Board (IRB) approval was required. Starting from a review of the literature, a research protocol was conceived and shared by the principal investigators (LC and FDM) with the steering committee. The protocol was peer-reviewed and published [17]. The leading references to create the protocol and the survey structure were gathered from Dal Mas et al. [18], Loftus et al. [19, 20], Cobianchi et al. [21], Venkatesh et al. [22], and Bashshur, Shannon and Sapci [23]. Before the initiative's official launch, the research protocol and the online survey were reviewed by the steering committee and filled in by a sample of surgeons to avoid mistakes.

The poll was made available at the end of November 2021 and was open until the middle of August 2022. All 917 WSES members received an e-mail invitation to participate in the survey. The initiative was also shared on the society's website and Twitter account. Additionally, the members of the Team Dynamics Study Group [14, 15] were also invited via e-mail. Four e-mail reminders were sent. WSES membership was not required to answer the survey. Still, it may be assumed that the vast majority of the participants come from the 917 WSES members to whom the research initiative was publicized, yielding an approximate 70% response rate.

The invitation e-mail included comprehensive details on the initiative's goals and rationale, the anticipated time frame (about 10 min), and the option to join the Team Dynamics Study Group to carry out additional research and disseminate the results. The identities of the participants were kept secret. Additionally, the identity of the investigators and the research protocol were kept private.

### Survey

The first group of questions aimed at understanding the participants’ features. The questions’ structure and list were derived from the previous Team Dynamics investigation [14, 15]. Surgeons were asked to disclose their gender, years of experience in trauma/emergency surgery, type of institution (academic versus non-academic), the country in which they work, role, eventual participation within a trauma team (institutionalized or not, and of which type), the kind of trauma leader, the educational training attended, and the eventual presence of diverse team members.

The second group of questions investigated the decision aids as reported by the surgical literature [18–20], by assessing the surgeons’ perception of 11 items using a 5-point Likert scale.

The third group of questions concerned the perceived challenges that surgeons need to face when making clinical decisions, assessing 13 items gathered from Loftus and colleagues [20] using a 5-point Likert scale.

The fourth section of the questionnaire aimed at assessing the surgeons’ feelings about AI by asking them if they were familiar with the term AI, their understanding of it through an open question, and the perceived importance of AI in surgery at the present time and in a five-year horizon. One last question aimed to assess the perceived benefits [21, 22] of the application of AI in surgery.

The survey’s questions related to clinical decision-making through new technological aids are reported in Appendix 1.

### Statistical analysis

The statistical analysis was conducted using the software R (RStudio 2022.07.0 + 548 "Spotted Wakerobin" Release) [24].

Manual coding was also employed concerning the qualitative questions. Surgeons were required to express their understanding of AI through an open question. Results were manually coded by two researchers (LC and FDM), who rated each statement as concordant, discordant, or inconclusive, following the analysis of Woltz et al. [25] and Cobianchi et al. [14].

## Results

### Participants

650 surgeons responded to the questionnaire. Located on five continents, participants hailed from 71 different nations. The sample, however, was not evenly dispersed, with the majority of surgeons coming from Europe (477, or 73%), particularly Italy (251, or 39%). 465 responders (72%) were from the 10 nations with the most participants overall.

118 female surgeons (18%), 531 male surgeons (82%), and one person who would rather remain anonymous made up the sample. Surgeons' years of experience in the field ranged from 1 to 35, with a mean of 12. The majority of participants (499, or 77% of the sample) were from academic institutions, and 540 of them declared to be part of a formally-established emergency surgery team (83%). Although there were a variety of roles indicated, senior consultants made up the bulk of surgeons (233, or 36%). Department heads made up 114 (18%) of the sample.

Table 1 presents descriptive statistics about the individuals and institutions that participated in the study, whereas Table 2 reports some information about the number of respondents according to their locations.

**Table 1:** Descriptive statistics about surgeons and institutions participating in the investigation

Item	Number of responses	% of total responses
Participants	650	100.00%
Males	531	81.69%
Females	118	18.15%
Prefer not to answer	1	0.15%

	Mean	SD
Years of experience	12.32	8.42
Minimum	1	
Maximum	36	

Kind of institution	Number	%
	650	100.00%
Academic	499	76.77%
Non-academic	151	23.23%

Role/position	650	100.00%
Senior Consultant	233	35.85%
Board-certified surgeon	179	27.54%
Resident	124	19.08%
Division chief or head	114	17.54%

Part of an emergency surgery team	650	100.00%
Yes	540	83.08%
No	110	16.92%

**Table 2:** Number of respondents according to their location

Total participants	650	100%
*Number of countries*	*71*	
*Continents*	*5*	*100%*
Europe	477	73%
Asia	85	13%
America	62	10%
Africa	22	3%
Oceania	4	1%

Ten most present countries	465	72%
Italy	251	39%
Greece	45	7%
Spain	37	6%
United Kingdom	37	6%
United States	26	4%
Turkey	16	2%
Malaysia	15	2%
France	14	2%
Brazil	13	2%
Ukraine	11	2%

### Clinical decision-making facilitators

Surgeons were given a list of 11 items referred to clinical decision-making facilitators to be rated on a 5-point Likert scale according to their perceived importance. Clinicians underlined the importance of training, clinical guidelines, and multidisciplinary committees and meetings, with a mean of over 4 out of 5. Interestingly, the most modern tools (namely, risk stratification by additive scores using static variable thresholds, machine learning and artificial intelligence, regression modeling and calculations—with 3.87, 3.56, and 3.49, respectively) received the lowest scores, with machine learning and artificial intelligence recording the highest standard deviation (1.07). The following Table 3 reports the results.

**Table 3:** Decision aids and facilitators

	Item	Mean	SD
1	Training	4.40	0.78
2	Clinical guidelines and cases	4.25	0.82
3	Multidisciplinary committees and meetings	4.19	0.85
4	Time spent to engage patients	4.10	0.89
5	Networking and international experiences	4.10	0.88
6	Non-technical skills	3.98	1.02
7	Mobile electronic medical records and online tools, including telemedicine	3.96	1.02
8	Publications	3.95	0.94
9	Risk stratification by additive scores using static variable thresholds	3.87	0.91
10	Machine Learning and Artificial Intelligence	3.56	1.07
11	Regression modeling and calculations	3.49	1.01

The responses given to the item “Machine Learning and Artificial Intelligence” were analyzed by dividing the sample by institution, position held, and country. Interestingly, the institution (academic or non-academic) and the role did not show any significant difference. Major differences emerged when analyzing countries, with some of them (like Argentina) showing a high mean (4.5) and a low standard deviation (0.548), and others (like Switzerland) a very low mean (2.6) and a high standard deviation (0.89). Such results are reported in the following Table 4.

**Table 4:** Item 10 “Machine Learning and Artificial Intelligence” responses per country

id	Country	# responses	Mean	SD
1	Argentina	6	4.5	0.548
2	Saudi Arabia	7	4.43	1.13
3	Brazil	13	4.38	0.768
4	Malaysia	15	4.13	1.30
5	India	8	4.12	0.835
6	France	15	4.07	0.884
7	United States	24	3.92	1.21
8	Ukraine	11	3.91	0.944
9	Bulgaria	9	3.89	0.928
10	Belarus	6	3.83	0.753
11	Russia	5	3.8	1.64
12	Spain	37	3.68	1.03
13	Netherlands	10	3.6	0.843
14	Germany	7	3.57	0.535
15	Romania	9	3.56	1.13
16	Turkey	16	3.5	0.966
17	Greece	45	3.42	1.14
18	Japan	5	3.4	0.548
19	Portugal	5	3.4	0.548
20	Italy	251	3.38	1.10
21	United Kingdom	37	3.35	1.09
22	Israel	6	3.17	0.408
23	Thailand	6	2.67	0.816
24	Canada	5	2.6	0.548
25	Switzerland	5	2.6	0.894

Moreover, the same responses were analyzed by considering the institution, position, and country simultaneously. Although numbers become smaller, some interesting findings emerge. For instance, all senior consultants belonging to Malaysian academic institutions firmly believed in AI, with all of them giving a rate of 5/5, compared to division chiefs working in the USA, who scored the item 2.5 with a standard deviation of 1.05. Despite being digital natives, residents do not appear in the top positions. Results are depicted in the following Table 5.

**Table 5:** Item 10 “Machine Learning and Artificial Intelligence” responses per institution, position, and country

id	Institution	Position	Country	#	Mean	SD
1	Academic	Senior-consultant	Malaysia	5	5	0
2	Academic	Division-chief	Brazil	7	4.57	0.535
3	Academic	Board-certified-surgeon	United-States	6	4.5	0.837
4	Academic	Senior-consultant	United-States	5	4.2	0.447
5	Academic	Board-certified-surgeon	France	6	4.17	0.753
6	Academic	Board-certified-surgeon	Greece	12	4	1.28
7	Academic	Division-chief	Ukraine	6	4	0.894
8	Academic	Resident	United-Kingdom	6	4	0.894
9	Academic	Senior-consultant	Bulgaria	5	4	1
10	Non-academic	Resident	United-Kingdom	8	3.88	1.13
11	Academic	Division-chief	Turkey	6	3.83	0.983
12	Academic	Resident	Greece	13	3.69	0.947
13	Academic	Senior-consultant	Romania	8	3.62	1.19
14	Academic	Board-certified-surgeon	Italy	45	3.6	1.18
15	Academic	Senior-consultant	Spain	20	3.6	0.995
16	Academic	Board-certified-surgeon	Malaysia	6	3.5	1.05
17	Non-academic	Senior-consultant	Italy	39	3.46	1.14
18	Academic	Board-certified-surgeon	Spain	11	3.46	1.13
19	Non-academic	Division-chief	Italy	14	3.43	0.756
20	Non-academic	Resident	Italy	5	3.4	0.548
21	Academic	Senior-consultant	Italy	46	3.35	1.06
22	Academic	Resident	Italy	56	3.34	1.16
23	Academic	Senior-consultant	Turkey	7	3.29	0.951
24	Non-academic	Senior-consultant	Greece	7	3.29	0.951
25	Academic	Senior-consultant	United-Kingdom	9	3.22	1.09
26	Academic	Division-chief	Israel	5	3.2	0.447
27	Non-academic	Board-certified-surgeon	Italy	29	3.17	1.04
28	Academic	Board-certified-surgeon	United-Kingdom	6	3.17	1.17
29	Academic	Division-chief	Italy	17	3.12	1.27
30	Academic	Senior-consultant	Greece	5	2.8	1.1
31	Academic	Division-chief	United-Kingdom	6	2.67	0.816
32	Non-academic	Board-certified-surgeon	Greece	5	2.6	0.548
33	Academic	Division-chief	United-States	6	2.5	1.05

### Challenges in clinical decision-making

Surgeons were asked to rate 13 items regarding the challenges that could arise when engaging in clinical decision-making. The items were adapted from Loftus et al. [20].

Results underline a perceived misalignment between the actual clinical scenario and the independent assessment, the lack of complete data availability, the risk of expecting specific outcomes grounded on inaccurate data, the presence of bias derived from the most recent experiences, and the possibility of making mistakes all along the clinical journey. Interestingly, surgeons rated the potential support granted by new technologies like AI as the lowest item (with a mean of 3.10 and the highest standard deviation of 1.14).

Findings are reported in the following Table 6.

**Table 6:** Challenges in clinical decision-making

	Item	Mean	SD
1	Sometimes the clinical scenario presented is different than the surgeon would have perceived during an independent assessment	3.61	0.95
2	Data are often incomplete	3.54	1.00
3	Patients are often informed of expected outcomes using data from aggregate patient populations without adjusting for their personalized risk profile	3.51	1.06
4	Recent experiences with a certain patient population or operation often affect disproportionately surgical decision-making than remote ones	3.50	0.98
5	Errors and mistakes are likely all along the way	3.49	1.09
6	Decisions must often be made before all relevant data can be retrieved	3.44	1.10
7	Potential outcomes are often predicted using personal beliefs rather than evidence-based guidelines	3.38	1.11
8	In-house calls happen often	3.35	1.00
9	It is often too complicated to form a complete list of all likely diagnoses, all life-threatening diagnoses, and all unlikely diagnoses that may be considered if the initial workup excludes other causes	3.34	1.04
10	A surgeon tends toward action when inaction may be preferable	3.28	1.07
11	The surgeon often falsely perceives that weaknesses and failures disproportionately affect their peers	3.17	1.01
12	It is often too complicated to recognize the strengths and limitations of available tests	3.12	1.02
13	Digital technologies (e.g., artificial intelligence) support how I take clinical decisions	3.10	1.14

### Knowledge and understanding of AI

Surgeons were asked if they were familiar with the term AI. 451 out of 650 participants (69% of the sample) declared they were familiar with the term, while 199 (31%) admitted they were not.

The following question required surgeons to write their understanding of AI applied to surgery. Each given statement was rated by the two principal investigators (LC and FDM) as concordant, discordant, or inconclusive.

To be rated as concordant, definitions needed to somehow stress the capability of the machine to mimic human intelligence or, at least, to express the aims and potential of AI applied to surgical practice or its technological functioning. Only 112 surgeons (17% of the sample) provided a statement that fitted the criterion. 178 participants (27% of the sample) gave responses that were incomplete, showing only a partial view of the phenomenon, being so rated as inconclusive. The remaining 360 surgeons (55% of the participants) gave answers that were not fitting the concept of AI, its aims, and its potential. Most of these participants declared they had no idea about what AI could entail in surgical practice.

The following Table 7 reports some examples of answers that were rated as concordant, inconclusive, and discordant [14, 25].

**Table 7:** Examples and ways of rating the given answers to the question: What is your understanding of AI applied to surgery?

Rated as	Given answer	Reason for rating
Concordant	“Capability of a machine to imitate intelligent human behavior.”	The statement recalls the ability of AI to mimic human behavior
	“It is capability of a computer system to mimic human cognitive functions.”	
	“A set of computer systems aiming at performing tasks normally based on human intelligence, starting from repetitive task and upscaling upon results.”	
	“Artificial intelligence is a technology which enables a machine to simulate human behavior. Machine learning is a subset of AI which allows a machine to automatically learn from past data without programming explicitly.”	
	“Artificial intelligence is the ability of a computer system to mimic human cognitive functions. Through AI, a computer system may simulate also the reasoning that people use to acquire new information and make decisions.”	
	“The ability of machines to learn complex data and then generate predictions can assist clinical decisions and which can be used to improve clinical care in surgery.”	The statement stresses the learning aspect and the capability to handle complex data to support decision-making
	“[AI] help[s] in the surgical daily practice obtained thanks to shared experience, obtained from several colleagues, throughout the collection of big data analyzed and optimized by means of computational analysis”	
	“AI/ML are not yet applicable in an extensive way to surgery. However, some relevant applications, particularly during the diagnostic phase (especially radiology), can influence surgical indication. After surgery, some examples of AI/ML algorithms are used in pathology to define the diagnosis better, thus the patient's prognosis.”	The statement shows a good understanding of the goals and potential of AI applied to surgery
	“[AI] works by combining large amounts of data with fast, iterative processing and intelligent algorithms, allowing software to automatically learn from data patterns or characteristics.”	The statement shows a good understanding of how the technology works
Inconclusive	“Application of big data.”	Although big data may be linked with the functioning of AI, the statement is incomplete
	“Simulation.”	Although simulations may be linked with the functioning of AI, the statement is incomplete
	“Telemedecine; robotic training.”	Although telemedicine/e-health and robotic surgery may be somehow linked with the functioning of AI, the statement is incomplete
	“Use of artificial intelligence for learning.”	The statement recalls only one scope, which is, by the way, not well identified
	“Technology using to facilitate decisions.”	
	“Useful for risk stratification and some decision-making.”	
	“It can help in making decisions and developing new techniques.”	
	“A predictive model”	
Discordant	“I don't have a deep understanding of how these work, although I have seen several publications on this lately.”	In the statements, participants admit they have poor or no knowledge about AI applied to surgery
	“I don’t know.”	
	“None.”	
	“Nothing.”	
	“I don't know AI applied to surgery.”	
	“Robotic surgery.”	Although the literature is starting to discuss AI-empowered surgical robots, the actual robotic surgical systems do not work with AI
	“Robots.”	
	“AI applied to surgical field.”	Redundant/repetition

The statements were also analyzed according to different keys, namely, the fact that they recalled the idea of AI supporting clinical decision-making and of learning/training, and the eventual other technologies linked to AI. Most surgeons (409, 63% of the sample) mentioned the impact on clinical decision-making, and 57 (9%) recalled the potential of AI for surgical training. Among the most cited technologies that may be linked with AI, there are Big Data (mentioned by 130 participants – 20% of the sample) and new technologies in general terms (107, 16%).

Table 8 highlights such results.

**Table 8:** Understanding of AI applied to surgery – Main results

Aims	#	%
Mentions the potential in supporting clinical decision-making	409	63
Mentions the potential in supporting learning/training activities	57	9
**Technologies linked with AI**		
Big data	130	20
New technologies in general	107	16
Robotics	22	3
Simulations	16	2
Virtual/augmented realities	8	1
Telemedicine	1	0
No other technologies mentioned	366	56
Total	650	

### The potential of AI today and tomorrow

Surgeons were asked to express their opinion about the importance of AI-based applications to support clinical decision-making at the current time and on a five-year horizon, using a 5-point Likert scale.

Participants rated the importance today with a mean of 3.06 out of 5 and a standard deviation of 1.1. In the future, the sample rated the importance as 3.88 as an average with a standard deviation of 0.97.

### The goals of AI

One last question referred to the perceived goals and benefits of AI-based in supporting clinical decision-making. Five items needed to be rated on a 5-point Likert scale.

Interestingly, surgeons rated all five items as equally relevant (ranging from a mean score of 3.51 to 3.78). The item with the highest score sees AI as a support tool to validate decisions that clinicians would make anyway.

The following Table 9 reports the results.

**Table 9:** Goals and benefits of AI applied to surgery

	Item	Mean	SD
1	It helps in evaluating/validating decisions I would take	3.78	1.01
2	It supports in taking complex clinical decisions;	3.73	1.10
3	It helps in scouting and reviewing publications;	3.70	1.03
4	It reduces the span of options;	3.53	1.01
5	It supports taking decisions regarding simple problems, so I can focus on high-value activities	3.51	1.10

## Discussion

The results of our survey are in line with previous studies [13], with trauma and emergency surgeons showing interest in AI but still having several doubts and concerns about its actual application and potential as a decision-making aid.

When enquired about the most effective tools to support clinical decision-making, surgeons declare to rely more on training, clinical guidelines, and the support of their multidisciplinary staff and colleagues. Interestingly, these three items represent central and “classical” elements in the trauma and emergency surgery context [14] and in the action of the leading scientific societies like the WSES, which promotes training modules for both physicians and nurses [26], clinical guidelines covering several aspects of the clinical profession [27], and studies on multidisciplinary team dynamics [14, 15]. AI and Machine Learning tools got one of the lowest rates (3.56), with the highest standard deviation (1.07). The broad standard deviation interval may represent a gap in the surgical community, with some surgeons firmly believing in the potential of such new technologies and others still having severe concerns about their practical application.

Interestingly, while no significant differences emerge when considering the institution and the position held, geographical differences occur in the sample, with some countries giving higher scores than others. It would be interesting to investigate further while such differences emerge, for instance, in terms of cultural mindset, training opportunities, availability of technology or tech partners, and presence of technological surgical leaders or ambassadors. Moreover, when analyzing the sample under different lenses simultaneously (institution, role, and country), residents do not appear to be technology enthusiasts and believers, despite their younger age and the definition of digital natives.

When considering the decision-making process dynamics, trauma and emergency surgeons underline the need for sound decision aids. Indeed, the most rated elements refer to the potential gap between reality and the surgeon's initial assessment and the lack of accurate data. Even in this case, most respondents did not believe that AI offers valuable support for decision-making. Like in the case of decision aids, the contribution of AI to decision-making got the highest standard deviation, depicting the two extreme views, with enthusiastic adopters in contrast with skeptics.

Regarding the knowledge about AI, while most surgeons declare to be familiar with the concept of AI, many exhibited only a partial understanding of AI. Interestingly, many surgeons recall big data technology, and some associate AI primarily with robotics. While robotic surgery may represent one promising application of AI in surgical science [13, 28, 29], AI in its current state offers sound decision-making support, like in the case of the POTTER algorithm as an emergency surgery risk calculator [5, 6].

The current general skepticism of trauma and emergency surgeons about AI is also confirmed by the given rate on the relevance of AI-based tools for decision-making as they appear today, with a mean of 3.06. One more time, a high standard deviation interval depicts divergent opinions and beliefs in the surgical community. Still, the future looks brighter, with a higher perceived relevance on a five-year horizon.

The same picture and the divergent trust in using AI-based decision tools emerge in the question about the goals and benefits. Interestingly, the degree of technology acceptance appears generally low, with surgeons being rather skeptical when it comes to AI ensuring better support and outcomes than traditional tools. Such a low degree of acceptance opens up new research avenues to investigate the main concerns in the practical adoption of AI or the ethical bias connected to it. In such a perspective, one primary concern may reside in the lack of technical knowledge regarding AI, which emerges from several questions, especially that on the understanding of AI. Moreover, when enquired about legal responsibility (a topic to which medical doctors are particularly sensitive), surgeons modestly rate the need to share their legal responsibility with either the manufacturer, those in charge of maintenance, or the data manager, and so the need to rethink the informed written consent when new technologies are involved.

### Limitations

Although our survey got a reasonably high response rate and 650 participants (approximately 70%), the sample is not equally distributed geographically. Indeed, most participants work in Europe and, more specifically, in Italy. The specific situation of the Italian and European contexts, including the actual access to AI-based technology and the medical education related to it, may have biased some of our results. Our limitations, along with the international community's perceived interest in AI [13], may stimulate new in-depth studies and investigations.

## Conclusion

In concluding our work, we return to the premises and research gaps that inspired it. AI-based applications are gaining traction in surgery as decision-making aids, and the surgical community is showing interest in them. Trauma and emergency contexts are often challenging, and surgeons perceive the need for sound decision-making tools.

Our results underline how the emergency surgical community seems to include surgeons who strongly believe in the potential contribution of AI technology and who may act as enthusiastic early adopters [19] and those who are reluctant, feeling more comfortable relying upon more classical aids like guidelines, traditional training, and multidisciplinary groups’ support. Some countries seem to have greater comprehension and trust of AI-based tools. Younger surgeons, who some presume to be keener on using new technologies, do not appear to have greater trust or understanding of AI compared with mid-career and senior surgeons.

Given the potential of AI in clinical practice [7, 20, 30] and the speed of development, the role of scientific societies like the WSES and surgical training programs are crucial to expanding knowledge regarding AI-enabled decision aids, disseminate new approaches, and encompass them in training modules and clinical guidelines, to bridge the gaps between technology enthusiasts and those who misunderstand and mistrust AI. In addition, AI-enabled decision aids in surgery should seek to gain the trust of surgeons by establishing transparency and by demonstrating performance advantages that improve patient care.

## Data Availability

The datasets used and/or analyzed during the current study are available from the corresponding author upon reasonable request.

## Abbreviations

AI: Artificial intelligence
WSES: World society of emergency surgery
CHERRIES: Checklist for reporting results of internet e-surveys
IRB: Institutional review board
SD: Standard deviation
