# Correction: Surgeons’ perspectives on artificial intelligence to support clinical decision-making in trauma and emergency contexts: results from an international survey

**DOI:** 10.1186/s13017-023-00493-9

**Published:** 2023-03-23

**Authors:** Lorenzo Cobianchi, Daniele Piccolo, Francesca Dal Mas, Vanni Agnoletti, Luca Ansaloni, Jeremy Balch, Walter Biffl, Giovanni Butturini, Fausto Catena, Federico Coccolini, Stefano Denicolai, Belinda De Simone, Isabella Frigerio, Paola Fugazzola, Gianluigi Marseglia, Giuseppe Roberto Marseglia, Jacopo Martellucci, Mirko Modenese, Pietro Previtali, Federico Ruta, Alessandro Venturi, Haytham M. Kaafarani, Tyler J. Loftus, Team Dynamics Study Group, Kenneth Lyle Abbott, Abubaker Abdelmalik, Nebyou Seyoum Abebe, Fikri Abu-Zidan, Yousif Abdallah Yousif Adam, Harissou Adamou, Dmitry Mikhailovich Adamovich, Ferdinando Agresta, antonino Agrusa, Emrah Akin, Mario Alessiani, Henrique Alexandrino, Syed Muhammad Ali, Vasilescu Alin Mihai, Pedro Miguel Almeida, Mohammed Mohammed Al-Shehari, Michele Altomare, Francesco Amico, Michele Ammendola, Jacopo Andreuccetti, Elissavet Anestiadou, Peter Angelos, Alfredo Annicchiarico, Amedeo Antonelli, Daniel Aparicio-Sanchez, antonella Ardito, Giulio Argenio, Catherine Claude Arvieux, Ingolf Harald Askevold, Boyko Tchavdarov Atanasov, Goran Augustin, Selmy Sabry Awad, Giulia Bacchiocchi, Carlo Bagnoli, Hany Bahouth, Efstratia Baili, Lovenish Bains, Gian Luca Baiocchi, Miklosh Bala, Carmen Balagué, Dimitrios Balalis, Edoardo Baldini, oussama Baraket, Suman Baral, Mirko Barone, Alberto Gonzãlez Barranquero, Jorge Arturo Barreras, Gary Alan Bass, Zulfu Bayhan, Giovanni Bellanova, Offir Ben-Ishay, Fabrizio Bert, Valentina Bianchi, Helena Biancuzzi, Chiara Bidoli, Raluca Bievel Radulescu, Mark Brian Bignell, Alan Biloslavo, Roberto Bini, Paolo Boati, Guillaume Boddaert, Branko Bogdanic, Cristina Bombardini, Luigi Bonavina, Luca Bonomo, Andrea Bottari, Konstantinos Bouliaris, Gioia Brachini, Antonio Brillantino, Giuseppe Brisinda, Maloni Mamada Bulanauca, Luis Antonio Buonomo, Jakob Burcharth, Salvatore Buscemi, Francesca Calabretto, Giacomo Calini, Valentin Calu, Fabio Cesare Campanile, Riccardo Campo Dall′Orto, Andrea Campos-Serra, Stefano Campostrini, Recayi Capoglu, Joao Miguel Carvas, Marco Cascella, Gianmaria Casoni Pattacini, Valerio Celentano, Danilo Corrado Centonze, Marco Ceresoli, Dimitrios Chatzipetris, Antonella Chessa, Maria Michela Chiarello, Mircea Chirica, Serge Chooklin, Christos Chouliaras, Sharfuddin Chowdhury, Pasquale Cianci, Nicola Cillara, Stefania Cimbanassi, Stefano Piero Bernardo Cioffi, Elif Colak, Enrique Colás Ruiz, Luigi Conti, Alessandro Coppola, Tiago Correia De Sa, Silvia Dantas Costa, Valerio Cozza, Giuseppe Curro’, Kirsten Felicia Ann-Sophie Aimee Dabekaussen, Fabrizio D’Acapito, Dimitrios Damaskos, Giancarlo D’Ambrosio, Koray Das, Richard Justin Davies, Andrew Charles De Beaux, Sara Patricia De Lebrusant Fernandez, Alessandro De Luca, Francesca De Stefano, Luca Degrate, Zaza Demetrashvili, Andreas Kyriacou Demetriades, Dzemail Smail Detanac, Agnese Dezi, Giuseppe Di Buono, Isidoro Di Carlo, Pierpaolo Di Lascio, Marcello Di Martino, Salomone Di Saverio, Bogdan Diaconescu, Jose J. Diaz, Rigers Dibra, Evgeni Nikolaev Dimitrov, Vincenza Paola Dinuzzi, Sandra Dios-Barbeito, Jehangir Farman Ali Diyani, Agron Dogjani, Maurizio Domanin, Mario D’Oria, Virginia Duran Munoz-Cruzado, Barbora East, Mikael Ekelund, Gerald Takem Ekwen, Adel Hamed Elbaih, Muhammed Elhadi, Natalie Enninghorst, Mairam Ernisova, Juan Pablo Escalera-Antezana, Sofia Esposito, Giuseppe Esposito, Mercedes Estaire, Camilla Nikita Farè, Roser Farre, Francesco Favi, Luca Ferrario, Antonjacopo Ferrario di Tor Vajana, Claudia Filisetti, Francesco Fleres, Vinicius Cordeiro Fonseca, Alexander Forero-Torres, Francesco Forfori, Laura Fortuna, Evangelos Fradelos, Gustavo P. Fraga, Pietro Fransvea, Simone Frassini, Giuseppe Frazzetta, Erica Pizzocaro, Maximos Frountzas, Mahir Gachabayov, Rita Galeiras, Alain A. Garcia Vazquez, Simone Gargarella, Ibrahim Umar Garzali, Wagih Mommtaz Ghannam, Faiz Najmuddin Ghazi, Lawrence Marshall Gillman, Rossella Gioco, Alessio Giordano, Luca Giordano, Carlo Giove, Giorgio Giraudo, Mario Giuffrida, Michela Giulii Capponi, Emanuel Gois, Carlos Augusto Gomes, Felipe Couto Gomes, Ricardo Alessandro Teixeira Gonsaga, Emre Gonullu, Jacques Goosen, Tatjana Goranovic, Raquel Gracia-Roman, Giorgio Maria Paolo Graziano, Ewen Alexander Griffiths, Tommaso Guagni, Dimitar Bozhidarov Hadzhiev, Muad Gamil Haidar, Hytham K. S. Hamid, Timothy Craig Hardcastle, Firdaus Hayati, Andrew James Healey, Andreas Hecker, Matthias Hecker, Edgar Fernando Hernandez Garcia, Adrien Montcho Hodonou, Eduardo Cancio Huaman, Martin Huerta, Aini Fahriza Ibrahim, Basil Mohamed Salabeldin Ibrahim, Giuseppe Ietto, Marco Inama, Orestis Ioannidis, Arda Isik, Nizar Ismail, Azzain Mahadi Hamid Ismail, Ruhi Fadzlyana Jailani, Ji Young Jang, Christos Kalfountzos, Sujala Niatarika Rajsain Kalipershad, Emmanouil Kaouras, Lewis Jay Kaplan, Yasin Kara, Evika Karamagioli, Aleksandar Karamarkovia, Ioannis Katsaros, Alfie J. Kavalakat, Aristotelis Kechagias, Jakub Kenig, Boris Juli Kessel, Jim S. Khan, Vladimir Khokha, Jae Il Kim, Andrew Wallace Kirkpatrick, Roberto Klappenbach, Yoram Kluger, Yoshiro Kobe, Efstratios Kofopoulos Lymperis, Kenneth Yuh Yen Kok, Victor Kong, Dimitris P. Korkolis, Georgios Koukoulis, Bojan Kovacevic, Vitor Favali Kruger, Igor A. Kryvoruchko, Hayato Kurihara, Akira Kuriyama, Aitor Landaluce-Olavarria, Pierfrancesco Lapolla, Ari Leppäniemi, Leo Licari, Giorgio Lisi, Andrey Litvin, Aintzane Lizarazu, Heura Llaquet Bayo, Varut Lohsiriwat, Claudia Cristina Lopes Moreira, Eftychios Lostoridis, Agustãn. Tovar Luna, Davide Luppi, Gustavo Miguel Machain V., Marc Maegele, Daniele Maggiore, Stefano Magnone, Ronald V. Maier, Piotr Major, Mallikarjuna Manangi, andrea manetti, Baris Mantoglu, Chiara Marafante, Federico Mariani, Athanasios Marinis, Evandro Antonio Sbalcheiro Mariot, Gennaro Martines, Aleix Martinez Perez, Costanza Martino, Pietro Mascagni, Damien Massalou, Maurizio Massaro, Belen Matías-García, Gennaro Mazzarella, Giorgio Mazzarolo, Renato Bessa Melo, Fernando Mendoza-Moreno, Serhat Meric, Jeremy Meyer, Luca Miceli, Nikolaos V. Michalopoulos, Flavio Milana, Andrea Mingoli, Tushar S. Mishra, Muyed Mohamed, Musab Isam Eldin Abbas Mohamed, Ali Yasen Mohamedahmed, Mohammed Jibreel Suliman Mohammed, Rajashekar Mohan, Ernest E. Moore, Dieter Morales-Garcia, MÃ¥ns Muhrbeck, Francesk Mulita, Sami Mohamed Siddig Mustafa, Edoardo Maria Muttillo, Mukhammad David Naimzada, Pradeep H. Navsaria, Ionut Negoi, Luca Nespoli, Christine Nguyen, Melkamu Kibret Nidaw, Giuseppe Nigri, Ioannis Nikolopoulos, Donal Brendan O’Connor, Habeeb Damilola Ogundipe, Cristina Oliveri, Stefano Olmi, Ernest Cun Wang Ong, Luca Orecchia, Aleksei V. Osipov, Muhammad Faeid Othman, Marco Pace, Mario Pacilli, Leonardo Pagani, Giuseppe Palomba, Desire’ Pantalone, Arpad Panyko, Ciro Paolillo, Mario Virgilio Papa, Dimitrios Papaconstantinou, Maria Papadoliopoulou, Aristeidis Papadopoulos, Davide Papis, Nikolaos Pararas, Jose Gustavo Parreira, Neil Geordie Parry, Francesco Pata, Tapan Patel, Simon Paterson-Brown, Giovanna Pavone, Francesca Pecchini, Veronica Pegoraro, Gianluca Pellino, Maria Pelloni, Andrea Peloso, Eduardo Perea Del Pozo, Rita Goncalves Pereira, Bruno Monteiro Pereira, Aintzane Lizarazu Perez, Silvia Pérez, Teresa Perra, Gennaro Perrone, Antonio Pesce, Lorenzo Petagna, Giovanni Petracca, Vorapong Phupong, Biagio Picardi, Arcangelo Picciariello, Micaela Piccoli, Edoardo Picetti, Emmanouil Pikoulis Pikoulis, Tadeja Pintar, Giovanni Pirozzolo, Francesco Piscioneri, Mauro Podda, Alberto Porcu, Francesca Privitera, Clelia Punzo, Silvia Quaresima, Martha Alexa Quiodettis, Niels Qvist, Razrim Rahim, Filipe Ramalho de Almeida, Rosnelifaizur Bin Ramely, Huseyin Kemal Rasa, Martin Reichert, Alexander Reinisch-Liese, Angela Renne, Camilla Riccetti, Maria Rita Rodriguez-Luna, Daniel Roizblatt, Andrea Romanzi, Luigi Romeo, Francesco Pietro Maria Roscio, Ramely Bin Rosnelifaizur, Stefano Rossi, Andres M. Rubiano, Elena Ruiz-Ucar, Boris Evgeniev Sakakushev, Juan Carlos Salamea, Ibrahima Sall, Lasitha Bhagya Samarakoon, Fabrizio Sammartano, Alejandro Sanchez Arteaga, Sergi Sanchez-Cordero, Domenico Pietro Maria Santoanastaso, Massimo Sartelli, Diego Sasia, NORIO SATO, Artem Savchuk, Robert Grant Sawyer, Giacomo Scaioli, DIMITRIOS SCHIZAS, Simone Sebastiani, Barbara Seeliger, Helmut Alfredo Segovia Lohse, Charalampos Seretis, Giacomo Sermonesi, Mario Serradilla-Martin, Vishal G. Shelat, Sergei Shlyapnikov, Theodoros Sidiropoulos, Romeo Lages Simoes, Leandro Siragusa, Boonying Siribumrungwong, Mihail Slavchev, Leonardo Solaini, gabriele soldini, Andrey Sopuev, Kjetil Soreide, APOSTOLOS SOVATZIDIS, Philip Frank Stahel, Matt Strickland, Mohamed Arif Hameed Sultan, Ruslan Sydorchuk, Larysa Sydorchuk, Syed Muhammad Ali Muhammad Syed, Luis Tallon-Aguilar, Andrea Marco Tamburini, Nicolò Tamini, Edward C. T. H. Tan, Jih Huei Tan, Antonio Tarasconi, Nicola Tartaglia, Giuseppe Tartaglia, Dario Tartaglia, John Vincent Taylor, Giovanni Domenico Tebala, Ricardo Alessandro Teixeira Gonsaga, Michel Teuben, Alexis Theodorou, Matti Tolonen, Giovanni Tomasicchio, Adriana Toro, Beatrice Torre, Tania Triantafyllou, Giuseppe Trigiante Trigiante, Marzia Tripepi, Julio Trostchansky, Konstantinos Tsekouras, Victor Turrado-Rodriguez, Roberta Tutino, Matteo Uccelli, Petar Angelov Uchikov, Bakarne Ugarte-Sierra, Mika Tapani Ukkonen, Michail Vailas, Panteleimon G. Vassiliu, Alain Garcia Vazquez, Rita Galeiras Vazquez, George Velmahos, Juan Ezequiel Verde, Juan Manuel Verde, Massimiliano Veroux, Jacopo Viganò, Ramon Vilallonga, Diego Visconti, Alessandro Vittori, Maciej Waledziak, Tongporn Wannatoop, Lukas Werner Widmer, Michael Samuel James Wilson, Sarah Woltz, Ting Hway Wong, Sofia Xenaki, Byungchul Yu, Steven Yule, Sanoop Koshy Zachariah, Georgios Zacharis, Claudia Zaghi, Andee Dzulkarnaen Zakaria, Diego A. Zambrano, Nikolaos Zampitis, Biagio Zampogna, Simone Zanghì, Maristella Zantedeschi, Konstantinos Zapsalis, Fabio Zattoni, Monica Zese

**Affiliations:** 1 Department of Clinical, Diagnostic and Pediatric Sciences, University of Pavia, Via Alessandro Brambilla, 74, 27100 Pavia, PV Italy; 2 General Surgery, IRCCS Policlinico San Matteo Foundation, Pavia, Italy; 3 ITIR – Institute for Transformative Innovation Research, University of Pavia, Pavia, Italy; 4 Department of Neurosurgery, ASUFC Santa Maria Della Misericordia, Udine, Italy; 5 Department of Management, Ca’ Foscari University of Venice, Venice, Italy; 6 Bufalini Hospital, AUSL Romagna, Cesena, Italy; 7 Department of Surgery, University of Florida Health, Gainesville, FL USA; 8 Division of Trauma and Acute Care Surgery, Scripps Memorial Hospital La Jolla, La Jolla, CA USA; 9 Department of HPB Surgery, Pederzoli Hospital, Peschiera del Garda, Italy; 10 General, Emergency and Trauma Surgery Department, Pisa University Hospital Pisa, Pisa, Italy; 11 Department of Economics and Management, University of Pavia, Pavia, Italy; 12 Department of Emergency, Digestive and Metabolic Minimally Invasive Surgery, Poissy and Saint Germain en Laye Hospitals, Poissy, France; 13 IRCCS Policlinico San Matteo Foundation, Pediatric Clinic., Pavia, Italy; 14 Department of Civil Engineering and Architecture, University of Pavia, Pavia, Italy; 15 Department of Surgery, Careggi University Hospital, Florence, Italy; 16 Humco Srl, Venice, Italy; 17 General Direction, ASL BAT (Health Agency), Andria, Italy; 18 Department of Political and Social Sciences, University of Pavia, Pavia, Italy; 19 Bureau of the Presidency, IRCCS Policlinico San Matteo Foundation, Pavia, Italy; 20 Harvard Medical School, Boston, MA USA; 21 Division of Trauma, Emergency Surgery, and Surgical Critical Care, Massachusetts General Hospital, Boston, MA USA

**Correction: World Journal of Emergency Surgery (2023) 18:1** **https://doi.org/10.1186/s13017-022-00467-3**

Following publication of the original article [1], in PubMed the author name Daniele Bissacco under Team Dynamics Study Group has not been tagged and now it has been rectified.

The original article has been corrected.

